# Anti-Fibrotic Effect of SDF-1β Overexpression in Bleomycin-Injured Rat Lung

**DOI:** 10.3390/pharmaceutics14091803

**Published:** 2022-08-27

**Authors:** Kleanthis Fytianos, Ronja Schliep, Sofia Mykoniati, Petra Khan, Katrin E. Hostettler, Michael Tamm, Amiq Gazdhar, Lars Knudsen, Thomas Geiser

**Affiliations:** 1 Department of Pulmonary Medicine, University Hospital Bern, 3010 Bern, Switzerland; 2 Department of Biomedical research, University of Bern, 3010 Bern, Switzerland; 3 Institute of Functional and Applied Anatomy, Hannover Medical School, 30625 Hanover, Germany; 4 Department of Internal Medicine, Cantonal Hospital of Jura, 2800 Delemont, Switzerland; 5 Department of Biomedical Research and Clinics of Respiratory Medicine, University Hospital Basel, University of Basel, 4031 Basel, Switzerland

**Keywords:** interstitial lung disease, IPF, lung fibrosis, SDF-1b, myofibroblast apoptosis, lung repair and regeneration, nonviral gene therapy, alveolar epithelial cell proliferation

## Abstract

**Rational:** Idiopathic pulmonary fibrosis (IPF) is a progressive interstitial lung disease and is associated with high mortality due to a lack of effective treatment. Excessive deposition of the extracellular matrix by activated myofibroblasts in the alveolar space leads to scar formation that hinders gas exchange. Therefore, selectively removing activated myofibroblasts with the aim to repair and remodel fibrotic lungs is a promising approach. Stromal-derived growth factor (SDF-1) is known to stimulate cellular signals which attract stem cells to the site of injury for tissue repair and remodeling. Here, we investigate the effect of overexpression of SDF-1β on lung structure using the bleomycin-injured rat lung model. **Methods:** Intratracheal administration of bleomycin was performed in adult male rats (F344). Seven days later, in vivo electroporation-mediated gene transfer of either SDF-1β or the empty vector was performed. Animals were sacrificed seven days after gene transfer and histology, design-based stereology, flow cytometry, and collagen measurement were performed on the tissue collected. For in vitro experiments, lung fibroblasts obtained from IPF patients were used. **Results:** Seven days after SDF-1β gene transfer to bleomycin-injured rat lungs, reduced total collagen, reduced collagen fibrils, improved histology and induced apoptosis of myofibroblasts were observed. Furthermore, it was revealed that TNF-α mediates SDF-1β-induced apoptosis of myofibroblasts; moreover, SDF-1β overexpression increased alveolar epithelial cell numbers and proliferation in vivo and also induced their migration in vitro. **Conclusions:** Our study demonstrates a new antifibrotic mechanism of SDF-1β overexpression and suggests SDF-1β as a potential new approach for the treatment of lung fibrosis.

## 1. Introduction

Idiopathic pulmonary fibrosis (IPF) is an age-related progressive lung disease with no curative therapy available [1]. IPF is associated with high mortality due to severe architectural destruction of the lung leading to respiratory failure with an average survival of 2–3 years after diagnosis [2,3,4]. Fibroblast activation, proliferation, invasion and differentiation to myofibroblasts in response to alveolar epithelial injury are the characteristics of active fibrogenesis [5,6]. Moreover, the histopathological hallmark of IPF lung is characterization by extensive alveolar scarring that contains fibroblasts and myofibroblasts [7]. Myofibroblasts are the main source of excessive extracellular matrix (ECM), including collagen, which contributes to organ failure [7]. Various cellular sources of myofibroblasts have been suggested and debated over the years. However, it is agreed that these cells contribute to the scar formation and are predominantly present in the fibroblastic foci [8,9,10,11,12]. Currently, there is no therapy capable of selectively eradicating myofibroblasts or reducing their production or propagation. Therefore, applying a method that can target myofibroblasts could be a key therapeutic strategy for treating patients with IPF. Stromal-derived growth factor-1 (SDF-1) or chemokine receptor ligand 12 (CXCL-12) is a cytokine and plays an important role in development and repair [13]. Increased local production of SDF-1 after injury has been reported to play important role in recruiting stem cells from the bone marrow at the site of injury to induce repair processes [14]. It has been reported in the past that SDF-1 regulates the migration of stem cells via its receptor CXCR4 and attenuates bleomycin-induced fibrosis in mice models [15]. Contrarily, there are other studies suggesting that SDF-1-dependent recruitment of CXCR4+ fibrocytes further aggravates the disease [16,17]. However, attempts were made to test CXCR4 antagonist as a potential antifibrotic agent that showed promising results in preclinical settings [18,19], but unfortunately could not be translated successfully into clinical application [20]. Interestingly however, SDF-1 mRNA has been alternatively spliced to generate splice variants, and total of five different variants have been described [21]. Importantly, most of the studies have so far only reported the effect of SDF-1α on migration and recruitment of bone marrow-derived cells. However, no study has been performed on other transcripts specifically to evaluate the effect of SDF-1β in vivo on the lung cells, and its role in fibrotic processes has not been studied. SDF-1β is very similar to SDF-1α and is most prominently present in cerebral microvessels; it is upregulated after cerebral ischemia, and responsible for the infiltration of CXCR4-expressing peripheral blood cells [22]. The present study sought to explore the effect of SDF-1β overexpression on various cells involved in the process of fibrogenesis using the bleomycin-injured rat lung model. Our results indicate that SDF-1β overexpression attenuates fibrosis in bleomycin-injured rat lungs by inducing myofibroblasts apoptosis; it also reduces collagen fibrils and total collagen and induces proliferation of alveolar epithelial cells. Together our data suggest that SDF-1β has direct antifibrotic effects that have not yet been explored.

## 2. Materials and Methods

### 2.1. Ethical Approval

Human lung tissue was obtained with the approval of the Human Ethics Committee of the University of Basel (Ref. Nr. EK: 05/06), Switzerland, and with the written informed consent of each patient.

Animal experiments were performed after approval from the local ethical committee, University and Cantone of Bern, Switzerland (Ref. Nr. BE/136-17).

### 2.2. Primary IPF Lung Fibroblasts 

IPF or control lung tissue pieces were placed into pre-wetted cell culture dishes containing DMEM/10%FCS (Cat. No. 32430-027, Thermo Fischer Scientific, Waltham, MA, USA). The cells grew by sprouting as described before [23] and were further passaged and used for experiments as described below.

### 2.3. Plasmid

SDF-1 (CXCL 12) transcript variant II (SDF-1β) (Origene, Rockville, ML, USA) was obtained and was inserted into minicircle plasmid (pSDF-1) obtained from Plasmid Factory GmbH, Bielefeld Germany. Subsequently, pSDF-1β was dissolved in endotoxin-free water and used for in vitro and in vivo application as described below.

### 2.4. In Vitro Transfection

IPF lung fibroblast transfection in vitro lung fibroblasts were transfected with either pSDF-1β or empty vector (EV) control plasmids via Lipofectamine^TM^ 3000 (Cat. No: L3000001, Thermo Fisher Scientific, Waltham, MA, USA) according to the manufacturer’s instructions. For TNF-α CRISPER/Cas9 KO plasmid ((sc-400124), Santacruz biotechnology, Dallas, TX, USA) transfection the cells were transfected using Nucelofactor (Lonza, Morristown, NJ, USA) following the parameters for fibroblast transfection as suggested. Supernatants were collected for cytokine measurements and cells were harvested for flow cytometry analysis or protein extraction. 

### 2.5. Wound-Healing Assay

Human alveolar adenocarcinoma cells (A549) (AATC, USA) from passages 5–15 were cultured in 6-well plates with fully supplemented RPMI1640 medium (Cat. No: 11875093, Thermo Fisher Scientific, Waltham, MA, USA). When confluent, with the use of a 200μL pipette tip, cells were gently scratched in the middle of the well. A549 cells were then treated with 10, 25, 50 and 100 ng/mL SDF-1β (Cat. No: 300-28B, BioConcept, Allschwil, Switzerland) with or without the presence of 6 μg/mL AMD 3100 octahydrochloride (TOCRIS, Cat. No: 3299, Bristol, UK), a highly selective CXCR4 receptor antagonist. Prior to SDF-1β treatment, the cells were treated with AMD 3100 for 20 min in order to inhibit the potential migratory effect of SDF-1. Light microscopy images were taken immediately after scratching (0 h) and after 24 h. The wound area was calculated with ImageJ software. Results are expressed as % of covered area. 

### 2.6. Animals

All animals received humane care in compliance with the ‘Principles of Laboratory Animal Care’ formulated by the National Society for Medical Research and the ‘Guide for the Care and Use of Laboratory Animals’ prepared by the Institute of Laboratory Animal Research. All experiments were performed in accordance with the standards of the European Convention of Animal Care.

### 2.7. Bleomycin Instillation

Schematic representation of the experimental setup is elaborated in Figure S1. On day 1 of the protocol, F344 rats (220–240 g) were anesthetized by inhalation of 4% isoflurane in a glass induction box, intubated with a 14-gauge intravenous catheter (Insyte, Madrid, Spain), and instilled intratracheally with bleomycin (Cat. No: 2634B5012, Baxter, Deerfield, IL, USA) (1.28 U/rat) in a volume of 500 μL to both lungs. Total animals studied where (*n* = 15) for stereological analysis *n* = 10 animals in each group were used and for other analysis *n* = 15 were used, 1 animal in the h-SDF-1 group died therefore *n* = 14 are reported.

### 2.8. pSDF-1 In Vivo Electroporation-Mediated SDF-1 Gene Transfer

Seven days after administration of bleomycin, the animals were randomly divided into two groups (*n* = 15 each). The first group received pSDF-1 plasmid 100 μg (concentration of 1 μg/mL) intratracheally and the second group received plasmid without insert (empty vector (EV)) followed by extracorporeal electroporation to both the lungs as described before [24] using NEPA 21 electroporator (Nepa Gene Co., Ltd., Ichikawa-City, Japan). Additional details are provided in the supplementary information. 

### 2.9. Assessment

All animals were sacrificed 14 days after bleomycin administration (7 days after pSDF-1 or EV gene transfer); animals were pre-anesthetized by inhaling 4% isoflurane, followed by the intraperitoneal administration of thiopental (50 mg/kg body weight). The heart–lung block was explanted, and tissue samples were collected for further analysis. Organs were harvested after a median thoraco-laparotomy, followed by flushing the pulmonary vasculature via the right ventricle with 0.9% sodium chloride solution. The right hilum was ligated, and the left lung was fixed by vascular perfusion fixation after two recruitment maneuvers at an airway opening pressure of 10 cm H_2_O on expiration. Fixation was performed using 1.5% glutaraldehyde, 1.5% paraformaldehyde in 0.15 M HEPES buffer and the left lung was used for design-based stereology, the right lung was used for hydroxyproline assay, ELISA, western blot and flow cytometry.

### 2.10. Hydroxyproline Assay

Resected lungs were weighted and frozen. The technique and all required reagents are as described before [22]. Results are expressed as μg of collagen/mg of wet lung weight.

### 2.11. Morphological Analyses at Light and Electron Microscopic Level

Lung volume was determined by fluid displacement, followed by a systematic uniform random sampling process [25]. Lung pieces assigned to light microscopic evaluation were embedded in glycol methacylate (Technovit 8100, Kulzer GmbH, Wehrheim, Germany), sectioned and stained with toluidine blue. A modified Ashcroft’s-score, ranging from 0–8, was used for a semi-quantitative scoring of pathological lesions. Between 20–30 fields of view per lung were randomly sampled and rated by an observer blinded to the samples based on published criteria [26]. At least 6 tissue blocks were sampled for electron microscopy and embed in epoxy resin. Ultrathin sections of a thickness of 60 nm were cut and subjected to a systematic uniform area sampling with a Morgagni 268 electron microscope (FEI, Eindhoven, The Netherlands). At a primary magnification of 8900, 20–30 images were randomly generated per section. These images were analyzed with the Stepanizer [27]. On every single image 16 points were projected and each point falling on the reference space (=the septal wall/parenchymatous lung tissue) was counted and assigned to the different structures of interest such as alveolar epithelial 1 and 2 (AEC1 and AEC2) cells, collagen fibrils, endothelial cells, capillary lumen, extra cellular matrix and cells being neither epithelial nor endothelial cell. These data were used to calculate the volume faction and the absolute volumes within reference space. The total volume of parenchymatous tissue/septal walls was determined at light microscopic levels using the newCAST stereology software for systematic uniform area sampling and projection of test points on the corresponding fields of view. At electron microscopy level, the basal lamina, covered by either AEC1 or AEC2 or injured cells were also determined, and surface fractions of basal lamina covered by these differently categorized epithelial cells were calculated. This was done using test-line segments and the Stepanizer for intersection counting of the test-line with the basal lamina covered either by alveolar epithelial type 1 cells (AEC1), alveolar epithelial type II cells (AEC2) or injured epithelial cells [28]. With these data, the septal surface covered with AEC1/AEC2 or injured epithelium was calculated.

### 2.12. Masson–Goldner Staining

Masson–Goldner staining was used to highlight connective tissue. The staining was performed on 4 µm thin tissue slices embedded in paraffin. For deparafinization slides were incubated in Xylol and the tissue was rehydrated in a descending ethanol concentration. The slides were incubated in hematoxylin followed by two washing steps in water and 1% acetic acid and transferred to azophloxin solution and washed again with 1% acetic acid. After incubation in phosphotungstic acid orange the slides were washed again with 1% acetic acid, followed by staining in light green solution and washing in 1% acetic acid. Finally, the slides were dehydrated using an ascending sequence of ethanol followed by a final incubation in Xylol and mounted.

### 2.13. Western Blot

A549 cells were grown to confluence and treated with different concentrations of recombinant human SDF-1 (10, 25, 50, 100 ng/mL) (Cat. No: 300-28B, BioConcept, Allschwil, Switzerland) in the absence or presence AMD (6 µg/mL). Either 15 min or 24 h after treatment cells were washed with ice-cold 1xPBS and lysed with Pierce lysis buffer (Cat. No: 87787, Thermo Fischer Scientific, Fisher Scientific, Waltham, MA, USA). The lysis procedure was performed on ice. Cell lysates were collected and centrifuged (300× *g*/15 min/4 °C). Protein content was measured with Bradford assay (Cat. No: 5000002, BIO-RAD, Hercules, CA, USA), and western blot was performed, using 7.5% gels (BIO-RAD, Cat. No.: 4561023, Hercules, CA, USA). Gels were run using Mini-Gel Trans Blot cell (BIO-RAD, Cat. No: 1704071) for 30 min at 60 mV and for 60 min at 120 mV and were transferred to Nitrocellulose membranes (BIO-RAD, Cat. No: 1704158) with the use of the Trans-Blot Turbo Transfer System (BIO-RAD, Cat. No: 17001918). The membranes were blotted using the PAK sampler kit (CST, Cat. No: 4750, Danvers, MA, USA). Visualization was done with a LI-COR Odyssey scanner. Western blot results were analyzed using Image Studio Lite Version 5.2 (LIQOR). Information regarding the antibodies used is given on Table S1. To block SDF-1 activity, in vitro experiments were performed using AMD3001 (Sigma Aldrich, St. Louis, MI, USA) at a dose of 6 µg/mL and effect was evaluated by western blot as described above. 

### 2.14. Flow Cytometry

Resected lungs were finely chopped into tiny pieces under sterile conditions with scissors and scalpel, and digested in collagenase solution as described in [29]. Cell suspensions were filtered through 100μm and 40μm cell strainers (Cat. No: 93,100 and 93040, SPL Life Sciences, Gyeonggi-do 487 835, Korea). Live, single-cell suspensions from rat lung homogenates were washed with PBS, counted, centrifuged (300× *g*/5 min/4 °C) and placed in 12 × 75 polypropylene tubes (Cat. No: 352053, BD Biosciences, Franklin Lakes, NJ, USA) at a seeding density of 500,000 cells/tube. Cells were re-suspended in 200 μL flow cytometry buffer (1X PBS, 5% BSA and 0.5% NaN_3_) and stained with surface or intracellular antibodies. A series of different antibody panels with different flourochroms was used as described in the SI section (Table S1). At least 10,000 live, single-cell events were recorded for each measurement in a BD LSR-II flow cytometer (BD bioscience, Franklin Lakes, NJ, USA). Data was analyzed with FlowJo software (Tree-Star, Ashland, OR, USA). Representative gating strategies for all experiments are shown on Figure S2.

### 2.15. ELISA

Human SDF-1 ELISA (Cat. No: E-EL-H0052, Elabscience, Wuhan, PRC) was used to measure human SDF-1 levels in rat lung homogenates. The rat SDF1/CXCL12 ELISA kit (Cat. No: LS-F23234-1, LS-Bioscience, Seattle, WA, USA) was used to measure SDF-1 levels in rat serum and lung homogenates; to measure TGF-β in rat lung homogenate, rat TGF-β ELISA (Cat. No: EK0514, Boster Bio, Pleasanton, CA, USA) was used. To study the anti-fibrotic mechanism of SDF-1 in vitro, TNF-α was measured in the supernatants of human IPF fibroblasts using TNF-α ELISA (Cat. No.: DY210-05, R&D Systems, Minneapolis, MN, USA). To measure TNF-α levels in rat lung homogenates and Broncho Alveolar Lavage (BAL), rat TNF-α ELISA (Cat. No: ab100785, Abcam, Cambridge, UK) was used. All assays were performed following manufacturer protocol.

### 2.16. Statistics

Prism 7 software (Graph Pad software Inc., San Diego, CA, USA) was used for statistics and for designing of the graphs. A parametric non-paired *t*-test was used.

## 3. Results 

### 3.1. Efficient Electroporation-Mediated pSDF-1β Gene Transfer in Bleomycin-Injured Rat Lungs

**Figure 1:**
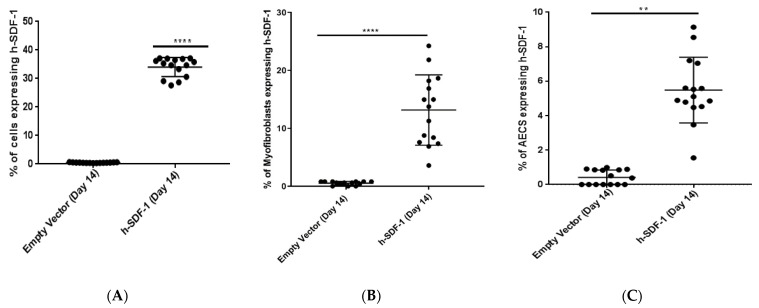
**Efficiency of electroporation-mediated h-SDF-1β gene transfer**. In vivo electroporation-mediated pSDF-1 gene transfer to bleomycin-injured rat lung is efficient. Total percentage of rat lung cells expressing hSDF-1 (**A**); percentage of, myofibroblasts (**B**), and alveolar epithelial cells (AEC) (**C**) expressing hSDF-1 seven days after pSDF-1 gene transfer. (*n* = 15); Mean ± SD; **: *p* < 0.01, ****: *p* < 0.0001).

Seven days after electroporation-mediated pSDF-1β gene transfer 34.04 ± 3.36% of total rat lung cells stained positive for hSDF-1β compared to 0.48 ± 0.11% in the Empty Vector group (EV) as measured by flow cytometry (Figure 1A). This corresponds with elevated hSDF-1β concentration, 30.68 ± 6.52 pg/mL as detected by ELISA in the lung homogenate in pSDF-1β-treated animals compared to 0.76 ± 0.16 pg/mL in EV (not presented as graph). Furthermore, to identify cell types that were transduced by pSDF1 in vivo, different cell surface markers were tested by flow cytometry. Interestingly, myofibroblasts (15.58 ± 1.3%) (Figure 1B) and alveolar epithelial cells (AEC) (5.82 ± 0.53%) (Figure 1C) stained for hSDF-1. Interestingly, no difference was observed in rat SDF-1 levels in both serum and lung homogenates between Empty Vector-and pSDF-1-treated animals (Figure S3B,C). 

### 3.2. pSDF-1 Gene Transfer-Reduced Total Collagen Content and Improved Histology in Bleomycin-Injured Rat Lung

**Figure 2:**
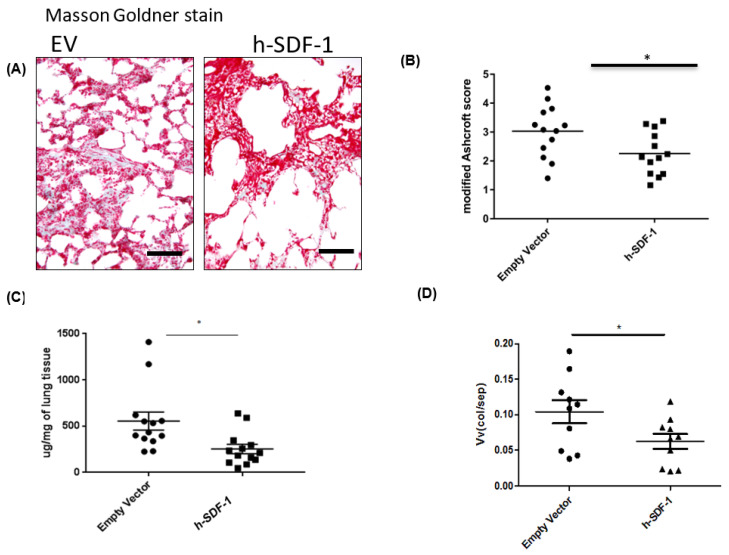
**Anti-fibrotic effect of h-SDF-1 overexpression.** In vivo electroporation-mediated pSDF-1 gene transfer reduces fibrosis in bleomycin-injured rat lung. Masson–Goldner staining of EV (**left**) and pSDF-1 (**right**) treated lung (**A**); assessment of lung fibrosis by modified Ashcroft’s score to grade fibrosis after pSDF-1 gene transfer (**B**); hydroxyproline assay to measure the total collagen content of rat lung after pSDF-1 or EV treatment (**C**); stereology-based assessment of volume fraction of collagen fibrils within septum/parenchyma of the rat lung tissue after pSDF-1 or EV treatment (**D**). (*n* = 15; Mean ± SD; *: *p* < 0.05) (1 animal in h-SDF-1 group died (*n* = 14)). (*n* = 10 for stereological analysis (**D**)).

Areas of collagen deposition were reduced after pSDF-1β gene transfer as compared to the EV group, as observed by Masson–Goldner staining (Figure 2A). Fibrosis was further quantified using a modified Ashcroft’s score and design-based stereology. Lung architecture was improved after pSDF-1β gene transfer compared to the empty vector as quantified by decrease in the modified Ashcroft’s score in hSDF-1β (2.26 ± 0.74) compared to EV (3.03 ± 0.90) (Figure 2B). Moreover, significant reduction in the collagen content, as assessed by hydroxyproline assay, in the animals treated with pSDF-1β (252.3 ± 50.06 vs. 554.5 ± 97.37 ug/mg of lung tissue) in EV-treated animals (Figure 2C) was observed. In accordance, a decrease in the volume fraction of collagen fibrils within the septum/parenchymatous tissue after pSDF-1β gene transfer (0.06258 ± 0.01050 vs. 0.1041 ± 0.01626), compared to EV was observed (Figure 2D). 

### 3.3. Composition of the Parenchymatous Lung Tissue after pSDF-1β Gene Transfer in Bleomycin-Injured Rat Lung

**Figure 3:**
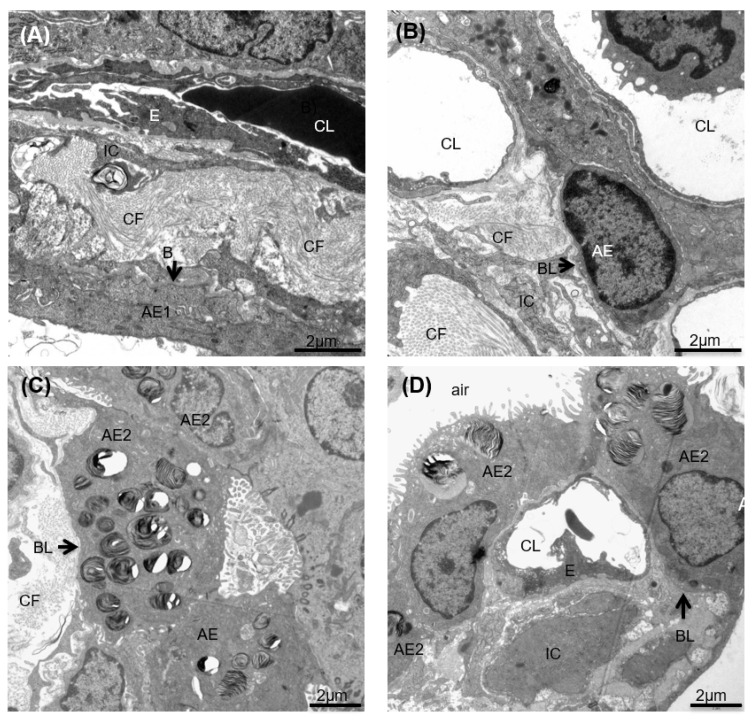
**Effect of SDF-1 over expression on ultrastructure of lung.** Electron micrograph of the bleomycin-injured lung treated with EV (**A**,**C**) and pSDF-1 (**B**,**D**). Accumulations of collagen fibrils (CF) could be seen in both groups. In addition, within the parenchymal tissue (=septal walls) alveolar epithelial cells (AE) could be distinguished from interstitial cells (IC), endothelial cells and the capillary lumen (CL). Hyperplasia of alveolar epithelial type II cells (AE2) could be identified in C and D. The alveolar epithelium covers the basal lamina (BL). These structures were further quantified in terms of their total volume within lung parenchyma using design-based stereology. Further abbreviations: E: endothelium; AE: alveolar epithelium.

**Figure 4:**
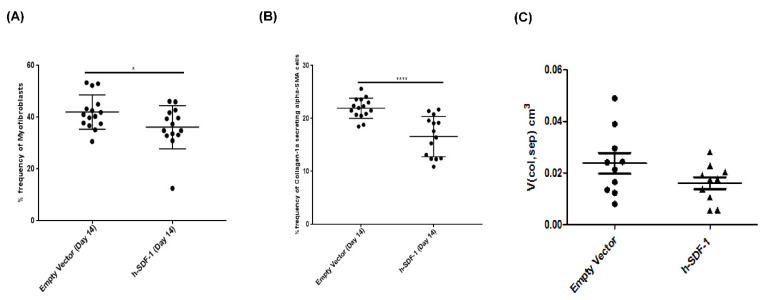
**Effect of h-SDF-1 transfection on collagen producing cells in vivo.** pSDF-1 gene transfer to bleomycin-injured rat lung reduced percentage of myofibroblasts (**A**) and collagen-producing cells (**B**), in accordance the volume of collagen fibrils in the parenchymal tissue was also reduced (**C**), compared to control animals. (*n* = 15; Mean ± SD; *: *p* < 0.05, ****: *p* < 0.0001). (1 animal in h-SDF-1 group died (*n* = 14)). (*n* = 10) for stereological analysis (**C**).

The samples were studied at electron microscopic level using design-based stereology to investigate the relative and absolute volumes of the different components forming lung parenchyma or interalveolar septa, respectively. Figure 3 demonstrates examples of the structures which could be differentiated. In both EV (Figure 3A,C) and pSDF-1 (Figure 3B,D) plenty of bundles of collagen fibrils could be detected with no clear difference between study groups. Also, hyperplasia of AEC2 cells was a common finding in both groups (Figure 3C,D). Since these parameters represented important readout parameters, a stereological, unbiased quantifications of the volumes of collagen fibrils with the parenchyma (Figure 2D and Figure 4C), as well as the surface area of the basal lamina covered by AEC2 (Figure 6B), were added. In addition, cellular structures were differentiated from the extracellular matrix (ECM). The cellular structures were further differentiated in alveolar epithelial cells (either alveolar epithelial type I (AEC1) or alveolar epithelial type II AEC2), endothelial cells and other cells (being neither endothelial nor epithelial cells). Regarding the ECM, collagen fibrils were further distinguished from the other components of the ECM, defined by ultrastructural criteria. No differences between the EV and the pSDF-1β were observed in the absolute volumes of cellular structures (0.1034 ± 0.009337 cm^3^ vs. 0.1267 ± 0.01054 cm^3^) or ECM (0.07948 ± 0.007051 cm^3^ vs. 0.07570 ± 0.003996 cm^3^). The volumes of AEC1 and AEC2 cells also showed no significant difference between the EV and the pSDF-1β groups (0.01331 ± 0.001659 cm^3^ vs. 0.01992 ± 0.003144 cm^3^ and 0.01176 ± 0.001860 cm^3^ vs. 0.01218 ± 0.001089 cm^3^). No changes were observed in the volume of the endothelium or the capillary lumen between the EV and pSDF-1 (0.02220 ± 0.002397 cm^3^ vs. 0.02657 ± 0.003397 cm^3^ and 0.05484 ± 0.01424 cm^3^ vs. 0.07521 ± 0.01472 cm^3^).

### 3.4. pSDF-1β Overexpression Reduces Collagen Producing myofibroblasts in the Bleomycin-Injured Rat Lung

Flow cytometry data shows a reduced percentage of myofibroblasts in pSDF-1β (36.09 ± 8.36% vs. 41.96 ± 6.62%), compared to EV-treated animals (Figure 4A). Similarly, α-SMA^+^ collagen secreting cells were reduced in pSDF-1 (16.59 ± 3.81% vs. 21.93 ± 1.92%) compared to EV-treated animals (Figure 4B). These findings are further complemented with the decrease in the total volume of collagen fibrils in the parenchymal tissue after treatment with pSDF-1 compared to the EV (0.01610 ± 0.002301 vs. 0.02375 ± 0.004006) (Figure 4C). However, TGF-β levels in lung homogenates did not show any difference in both animal groups (Figure S3A). 

### 3.5. pSDF-1β Overexpression Induces Apoptosis in the Myofibroblasts in the Bleomycin-Injured rAT Lung

**Figure 5:**
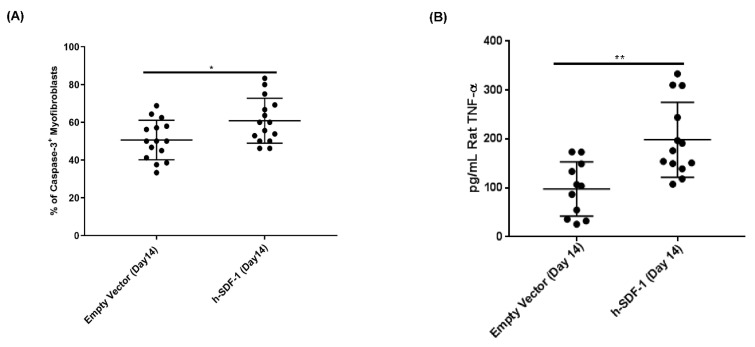
**h-SDF-1 overexpression induces apoptosis on myofibroblasts in vivo.** pSDF-1 gene transfer to bleomycin-injured rat lung induces apoptosis of myofibroblasts. Increased caspase-3-positive cells after pSDF-1 treatment (**A**), SDF-1 induces apoptosis by TNF-α-mediated pathway; increased TNF-α in rat lung homogenate after pSDF-1 treatment (**B**). *: *p* < 0.05, **: *p* < 0.01.

Caspase 3-dependent apoptosis in myofibroblasts is induced in pSDF-1 treated group compared to the EV group (60.83 ± 11.92% vs. 50.6 ± 10.49%), (Figure 5A). Interestingly, level of TNF-α was significantly increased after SDF-1β transduction 198.50 ± 21.26 pg/mL vs. 97.89 ± 16.69 pg/mL in the empty vector control group (Figure 5B).

### 3.6. pSDF-1β Overexpression Increases Number of Alveolar Epithelial Cells, Increases the Epithelial Basal Lamina Covering Epithelial Cells and Stimulates Proliferation of Alveolar Epithelial Cells in the Bleomycin-Injured Rat Lung

**Figure 6:**
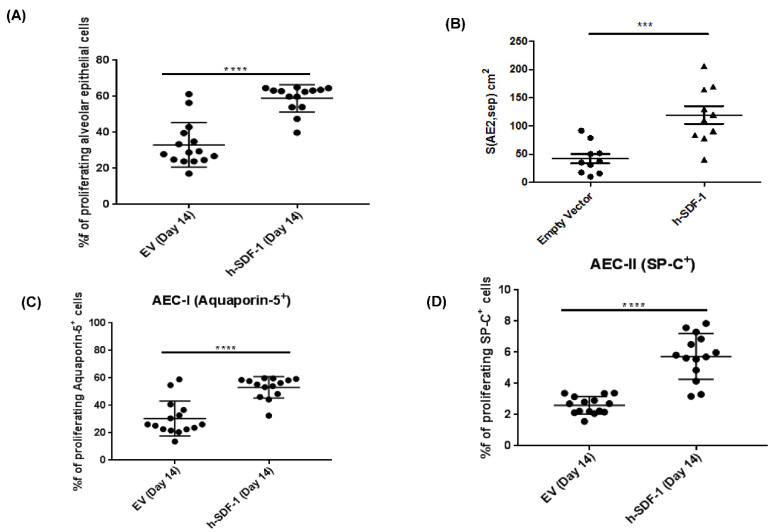
**h-SDF-1 overexpression induces AEC proliferation.** Compared to the control, pSDF-1 gene transfer to bleomycin-injured rat lung increases proliferation of alveolar epithelial cells (AEC). Flow cytometry data revealed an increased percentage of total alveolar epithelial cells; (**A**) furthermore, the percentage of proliferating AEC type I (**C**) and proliferating AEC II (**D**) were also increased. Moreover, the total septal surface area, defined by the epithelial basal lamina, and covered with AEC2 cells was significantly increased (**D**). (*n* = 15) (Mean ± SD; ****: *p* < 0.0001, ***: *p* < 0.001). (1 animal in h-SDF-1 group died (*n* = 14)). (*n* = 10) for stereological analysis (**B**).

Overexpression of pSDF-1β increased epithelial cells (58.91 ± 2.01% vs. 33.06 ± 3.19%) compared to EV group (Figure 6A). This is further supported by calculating the total septal surface area, defined by the epithelial basal lamina, covered with AEC2 cells that was significantly increased after hSDF-1β treatment compared to the EV (119.3 ± 15.77 cm^2^ vs. 41.87 ± 8.47 cm^2^), (Figure 6B). Complementary to this, pSDF-1β transfection significantly increased proliferation of both AEC type I (53.17 ± 7.83% vs. 30.45 ± 12.67%) and AEC type II (5.73 ± 1.46% vs. 2.60 ± 0.56%) compared to EV transfection as shown by flow cytometry (Figure 6C,D). 

### 3.7. pSDF-1β Induces TNF-α-Mediated Apoptosis in IPF Lung Fibroblasts In Vitro

**Figure 7:**
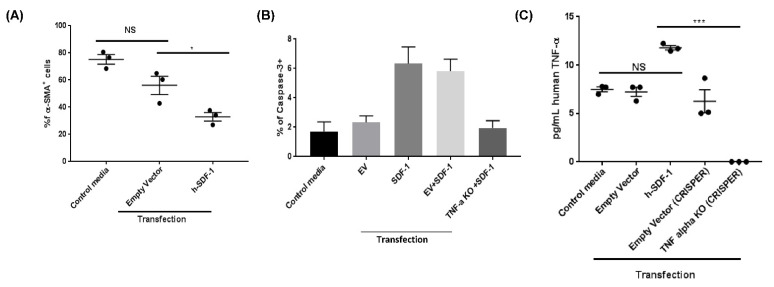
**Effects of h-SDF-1 in vitro.** Increased apoptosis of IPF lung fibroblasts after pSDF-1 transfection in vitro (**A**), the apoptosis is Caspase 3-mediated (**B**) and TNF-α plays role in induction of apoptosis (**C**). (*n* = 3, *: *p* < 0.05; ***: *p* < 0.001, NS (not significant)).

In vitro experiments on human IPF lung fibroblasts show that pSDF-1β transfection significantly decreased number of α-SMA^+^ cells (32.93 ± 3.12 vs. 56.08 ± 6.75 (Figure 7A), and increased frequencies of apoptotic α-SMA^+^ cells (6.047 ± 1.53 vs. 2.39 ± 0.60, *p* < 0.05) (Figure 7B) compared to EV transfection. The empty vector alone also showed slight reduction in α-SMA^+^ cells and slight increase in Caspase3^+^ cells; this could be the effect of transfection and also the small sample size. Moreover, increased TNF-α levels were observed in the cell culture supernatants of pSDF-1β-transfected cells compared to the control cells (11.8 ± 0.22 vs. 7.23 ± 0.47 pg/mL) (Figure 7C). Furthermore, when IPF fibroblasts were transfected with TNF-α CRISPR/Cas9 KO plasmid, followed by pSDF-1β transfection, the frequency of caspase-3-positive cells was significantly reduced (Figure 7D).

### 3.8. pSDF-1 Improves In Vitro Alveolar Epithelial Wound Repair via the PAK 2 Pathway

**Figure 8:**
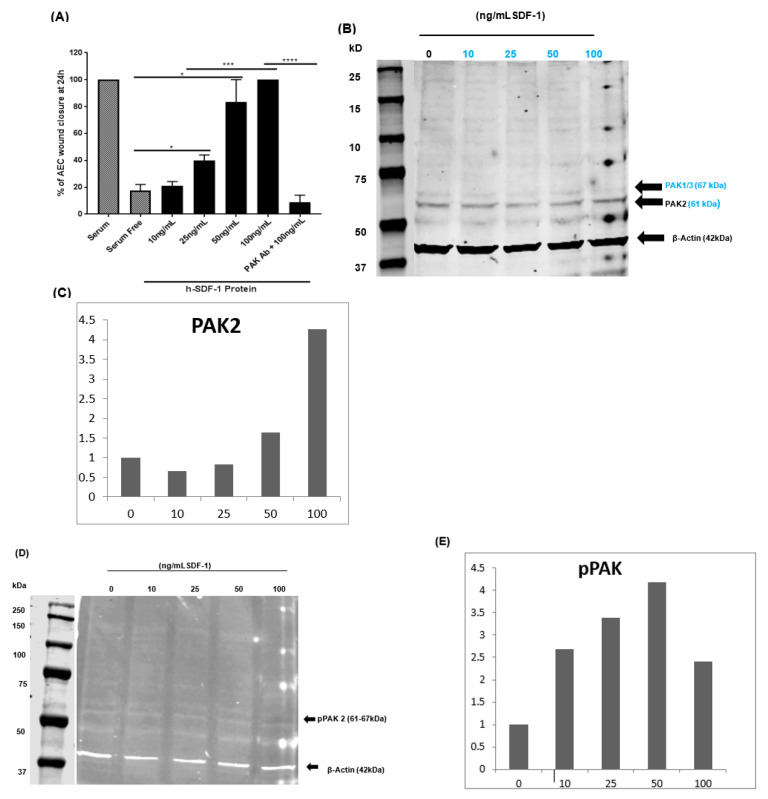
SDF-1 increases alveolar epithelial cell wound healing and induces migration via PAK pathway. Alveolar epithelial cells’ (A549) wound healing was improved after SDF-1 treatment in a dose-dependent manner in in vitro scratch assay (**A**). A549 cells treated with SDF-1 western blot analysis revealed increased PAK 2 in dose-dependent manner (**B**–**D**). Moreover, the phosphorylation of PAK was also observed after SDF-1 treatment (**E**). (Mean±SEM *: *p* < 0.05, ***: *p* < 0.001, ****: *p* < 0.0001).

In vitro wound-healing experiments were performed on A549 cells. Recombinant SDF-1 was used at different concentrations. Improved wound-healing in a concentration-dependent manner was observed with the strongest effect at 100 ng/mL (100 ± 0% vs. 14.60 ± 2.59%) compared to control group (Figure 8A). Interestingly, AMD 3100 significantly reduced wound-healing capacity of SDF-1 (Figure S5A). Moreover, PAK 2 and pPAK 2 activities was increased after treatment with SDF-1 in a dose-dependent manner (Figure 8B,C). Moreover, neutralization of PAK in presence of 100 ng/mL SDF-1 significantly reduced wound closure compared to SDF-1 (100 ng/mL)-treated cells (100 ± 0% vs. 8.76 ± 5.30%) (Figure 8A). Also, treatment with AMD 3100, resulted in reduced PAK 2 and pPAK 2 signal intensity on western blot gels (Figure S5B–E). 

## 4. Discussion

SDF-1β overexpression attenuated lung fibrosis, reduced total collagen content, induced apoptosis of collagen-producing myofibroblast and increased proliferation of alveolar epithelial cells in the bleomycin-injured rat lung model. We also observed that myofibroblast apoptosis was mediated via TNF-α, and in vitro alveolar epithelial cell migration was regulated via PAK 2 after SDF-1β treatment. 

We have recently reported the presence of stem cells of mesenchymal origin (MSC) in fibrotic lung [22,30], and speculated that although these cells are present they are not sufficient to stimulate or help in repair process. Therefore, we overexpressed SDF-1β to the injured lung using in vivo electroporation-mediated gene transfer [24] with the aim to increase the recruitment of MSC to the lung to facilitate repair. SDF-1 is ubiquitously expressed and is regulated at splicing. Although SDF-1α is the most predominant variant, it is however easily degraded after entering in the blood [31]. Since the current study is a translational project we choose human transcript to evaluate its effect, moreover the Rat SDF-1 is highly conserved, with >95% identity to its known human counterpart [32]. We used SDF-1β variant, although SDF-1β isoform is more limited in availability; however, due to its activity in the blood it seems to be potent for inter-organ communication via blood-mediated gradients [31]. As suggested before in an in vitro migration assay [15], we observed no increase in the number of MSC- or CXCR4-positive cells in response to SDF-1β overexpression in the injured lung. However, we observed that myofibroblast number was reduced and in accordance the total collagen was reduced. Further detailed stereology also showed a reduced fraction of collagen fibrils in the lung parenchyma compared to the control lung. These findings were interesting in relation to lung fibrosis. Notably, we identified caspase-3-mediated apoptosis of myofibroblasts after SDF-1β treatment in vivo mediated by TNF-α. Activation and nuclear localization of Caspase-3 by TNF-α has been suggested in relation to adipocytes [33], and TNF-α-dependent apoptosis by SDF-1 has been reported in cardiac myocytes [34]. Interestingly, the role of proinflammatory cytokine TNF-α in lung fibrosis has been investigated with contradictory reports, two studies showing permanent expression of TNF-α by alveolar epithelial cells leading to fibrotic lesions in the mice lung [35,36]. However, a recent study suggests that TNF-α can resolve lung fibrosis by targeting the profibrotic macrophages in the rodent model of lung fibrosis [37]. In this study by Redente EF et al. [37], authors report that TNF-α is anti-fibrotic in established fibrosis. Interestingly, however in early stages of fibrosis in the bleomycin-injured mice lung TNF-α levels are upregulated during the inflammatory phase; however, these levels subside when fibrosis is established [38]. These findings indicate stage-dependent pleotropic expression and the role of TNFα in course of lung fibrosis in animal model. Although SDF-1 plays an essential role in homing and recruitment of bone marrow stem cells, there are controversies over the protective versus apoptotic effects of SDF-1/CXCR4 axis in injury models, with most studies reporting SDF-1-induced apoptosis [39,40,41]. In our study, we observed increased TNF-α levels in response to SDF-1 overexpression in established fibrosis, and showed TNF-α-mediated apoptosis in line with previous studies. In the bleomycin injury model, we transduced SDF-1β using minicircle plasmid that is ubiquitous, and observed that diverse cell types including macrophages, fibroblasts, myofibroblast and alveolar epithelial cells in the rat lung expressed human SDF-1β at day 7 after electroporation-mediated gene transfer. Our finding indicates that the effect we have on myofibroblasts could probably be an additive paracrine effect. To further confirm these findings, we used lung fibroblasts obtained from patients suffering from IPF and transfected them either with SDF-1β or control plasmid and observed similar findings as in vivo; that is, a reduced number of myofibroblasts, increased caspase 3-positive myofibroblasts and a relative increase of TNF-α after SDF-1β transfection in vitro. To further support our findings, we silenced TNFα in the IPF lung fibroblasts using CRISPER Knock Out plasmid and observed reduced apoptosis even in presence of SDF-1β overexpression in vitro, confirming a TNFα-dependent mechanism. These findings indicate that SDF-1β has a similar direct effect on myofibroblast as seen in the bleomycin injury model. Most crucial aspect of IPF pathophysiology is the failure of alveolar epithelium to heal in response to multiple injuries [42]. Therefore, an early and orderly reepithelization of injured alveolar epithelium is very essential for repair and regeneration of injured lungs. SDF-1 has been shown to play a role in intestinal epithelial cell-spreading and migration [43] skin wound-healing [44] and migration of corneal epithelial cells [45]. Interestingly, there is a report suggesting impaired alveolar epithelial cell migration and reduced lung-healing due to low SDF-1 in patients that developed pulmonary airway leak (PAL) following lobectomy [46]. We observed increased alveolar epithelial cells seven days after SDF-1β gene transfer in bleomycin-injured rat lung; this finding was further supported by an increase in the total surface area, of the epithelial basal lamina that is covered with alveolar epithelial type II cells as measured by stereological method. Interestingly, we could also observe increased proliferation of epithelial cells that stain for aquaporin 5 (alveolar epithelial type I) and for surfactant protein C (alveolar epithelial type II) cells in contrast to what is reported [46]. To know if the beneficial effect is by epithelial cell proliferation only or SDF-1β also promotes migration, we performed in vitro wound-healing assay and observed dose-dependent improvement in the wound healing of alveolar epithelial cells in response to SDF-1β treatment. Furthermore, we observed that SDF-1β treatment induced migration via activation of PAK pathway in our in vitro wound-healing assay as suggested before [47]. We observed increased PAK 2 activity and phosphorylation in response to SDF-1β treatment suggesting actin polymerization leading to cell migration. PAK 2 has also been reported to promote proliferation and motility of cancer cells [48]. We have simultaneously observed two contrasting phenomena: firstly that SDF-1 overexpression inhibited the generation of myofibroblasts; secondly, we have also found that the proliferation and migratory ability of alveolar epithelial cells was increased after SDF-1 overexpression. Myofibrobalst apoptosis is a key antifibrotic process, however migration of alveolar epithelial cells might confuse as a first step towards epithelial meschencymal transition (EMT), a complex process that transforms epithelial cells to myofibroblasts and contribute to fibrosis [49]. However, proliferation of alveolar epithelial cells along with migration indicates that SDF-1 helps in alveolar reepithelization rather than EMT. Moreover, contrary effects of SDF-1 on different cell types indicates its broad spectrum of mechanisms that can balance the epithelial and mesenchymal paradox which is the key regulator in lung fibrosis [50], thus making SDF-1 an interesting antifibrotic agent. Recently, SDF-1 gene transfer to infracted myocardium was reported to be safe and improved heart failure symptoms in patients with ischemic cardiomyopathies [51], this gives us confidence that SDF-1 gene transfer can also be applied for patients suffering from lung fibrosis. Current proof of concept study is limited by the fact that we have only tested one-time point of day seven after gene transfer; long term studies should be evaluated to see the beneficial effect of SDF-1 gene transfer in bleomycin-induced lung fibrosis. Although we performed elaborate morphometric analysis to see the effect of SDF-1 gene transfer on the lung architecture and explored novel mechanism of myofibroblast apoptosis, other yet unknown mechanisms must be investigated for successful clinical translation of SDF-1. Targeting profibrotic myofibroblasts as a therapeutic approach to the treatment of pulmonary fibrosis has been suggested and tested [52]; however, our data for the first time demonstrates antifibrotic effects of SDF-1β overexpression, not only by affecting myofibroblasts but also by inducing alveolar epithelial cell proliferation, therefore making it a very promising candidate for lung remodeling and treatment of IPF.

## Data Availability

Not applicable.

## Supplementary Materials

The following supporting information can be downloaded at: https://www.mdpi.com/article/10.3390/pharmaceutics14091803/s1, Figure S1: Schematic representation of the animal experimentation. Male F344 rats (220–240g) were used. Initially, 1.28U/g of bleomycin was instilled intra-tracheally. 7 days after bleomycin instillation, either h-SDF-1 or EV in vivo mediated electroporation took place. Rats were sacrificed either on Day 9 (2 days post-transfection) or at Day 14 (7 days post-transfection), in order to allow comparisons between early and late time points post-transfection. For the h-SDF-1 and EV-transfected groups 15 animals per group were used. 5 untreated rats were used as controls. A total of 35 rats were used; Figure S2: Flow cytometry gating strategies. Representative gating strategies for all cell types and measured end-points are shown. Lists of the antibodies that were used and technical specifications are shown on Table S1. More specifically: (A) Gating strategy for rat and human SDF-1 secreting cells. (B) Gating strategy for MSCs and CXCR4/SDF-1 expression. (C) Gating strategy for myofibroblasts and collagen-1a α-SMA cells. (D) Gating strategy for apoptotic myofibroblasts. (E) Gating strategy for epithelial cells and proliferating epithelial cells. (F) Gating strategy for IPF fibroblasts expressing both α-SMA and Caspase-3. (G) Gating strategy for cell specific hSDF-1 secretion. For all figures except (F), cells obtained from rat lung homogenates were used. For (F) cells from IPF patients were used. For each sample, at least 10,000 live, single events were recorded at an LSR-II BD FACS instrument; Figure S3: Rat TGF-β and rat SDF-1 measurements in vivo. (A) Rat TGF-β levels. (B) Rat SDF-1 levels in lung homogenates and (C) Serum. ELISA measurements were used for quantification of rat TGF-β and rat SDF-1 (*n* = 15). No significant differences are observed. Rat SDF-1 expression (D) Percentage of cells expressing rat SDF-1 seven days after gene transfer. (E) Number of MSCs in the lung 7 days after SDF-1 gene transfer were not increased compared to the control group; Figure S4: pSDF-1 transfection of IPF fibroblasts after TNF-α CRISPER/Cas9 KO transfection did not induce production and release of TNF-α as measured in cell culture supernatant (A) and in cell extract (B) by ELISA; Figure S5: Inhibitory effect of AMD to SDF-1 in regards to wound healing capacity and PAK expression. For the wound healing and Western blot experiments, A549 cell cultures were treated for 20 min with 6 μg/mL of AMD, a CXCR4 receptor antagonist. The h-SDF-1 concentrations are shown on X-axis. Cells were initially treated with AMD and then with h-SDF-1. (A) AMD inhibits the effect of SDF-1 on epithelial cell migration. Wound Healing assay for A549 cells (*n* = 3, Error bars: Mean ± SEM). Regarding PAK expression, Western blot was performed. (B) PAK1/2/3 expression. (C) Quantification of PAK1/3 signal. (D) pPAK expression. (E) Quantification of PAK2; Table S1: Flow cytometry and Western blot antibodies. The list includes all antibodies that used for the flow cytometry and Western blot experiments with all technical specifications.

## Abbreviations

IPF: Idiopathic pulmonary fibrosis; SDF1: Stromal derived growth factor; CXCL-12: chemokine receptor 12; CXCR-4:C-X-C chemokine receptor type; TNF-α: Tumor necrosis factor; pSDF-1: plasmid stromal derived growth factor; EV: Empty Vector; CRISPR/Cas9: clustered regularly interspaced short palindromic repeats/CRISPR associated protein 9; AEC1: alveolar epithelial type 1 cells; AEC2: alveolar epithelial type II cells; PAK 2: The p21-activated kinase.
