# Supplementary material for: Anti-Fibrotic Effect of SDF-1β Overexpression in Bleomycin-Injured Rat Lung
Source: Pharmaceutics. 2022 Aug 27;14(9):1803. doi: 10.3390/pharmaceutics14091803 (PMC9502331; doi:10.3390/pharmaceutics14091803)
Supplement: Supplementary file 1 [file pharmaceutics-14-01803-s001.zip › pharmaceutics-1865357-supplementary.pdf]

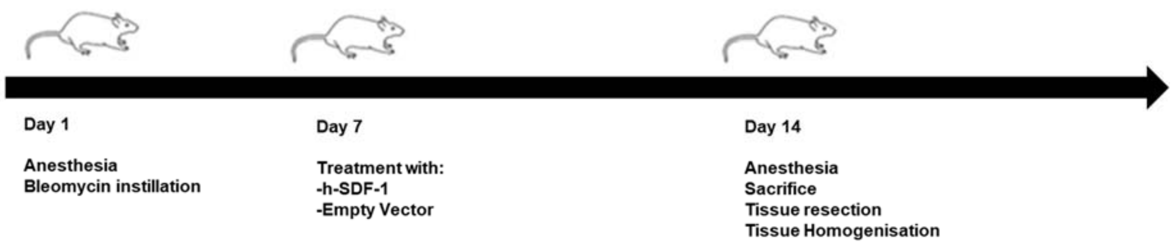

Figure S1. Schematic representation of the animal experimentation. Male F344 rats (220-240g) were used. Initially, 1.28U/g of bleomycin was instilled intra-tracheally. 7 days after bleomycin instillation, either h-SDF-1 or EV in vivo mediated electroporation took place. Rats were sacrificed either on Day 9 (2 days post-transfection) or at Day 14 (7 days post-transfection), in order to allow comparisons between early and late time points post-transfection. For the h-SDF-1 and EV-transfected groups 15 animals per group were used. 5 untreated rats were used as controls. A total of 35 rats were used.

Gating strategy for rat and human SDF-1secreting cells (In vivo experiments

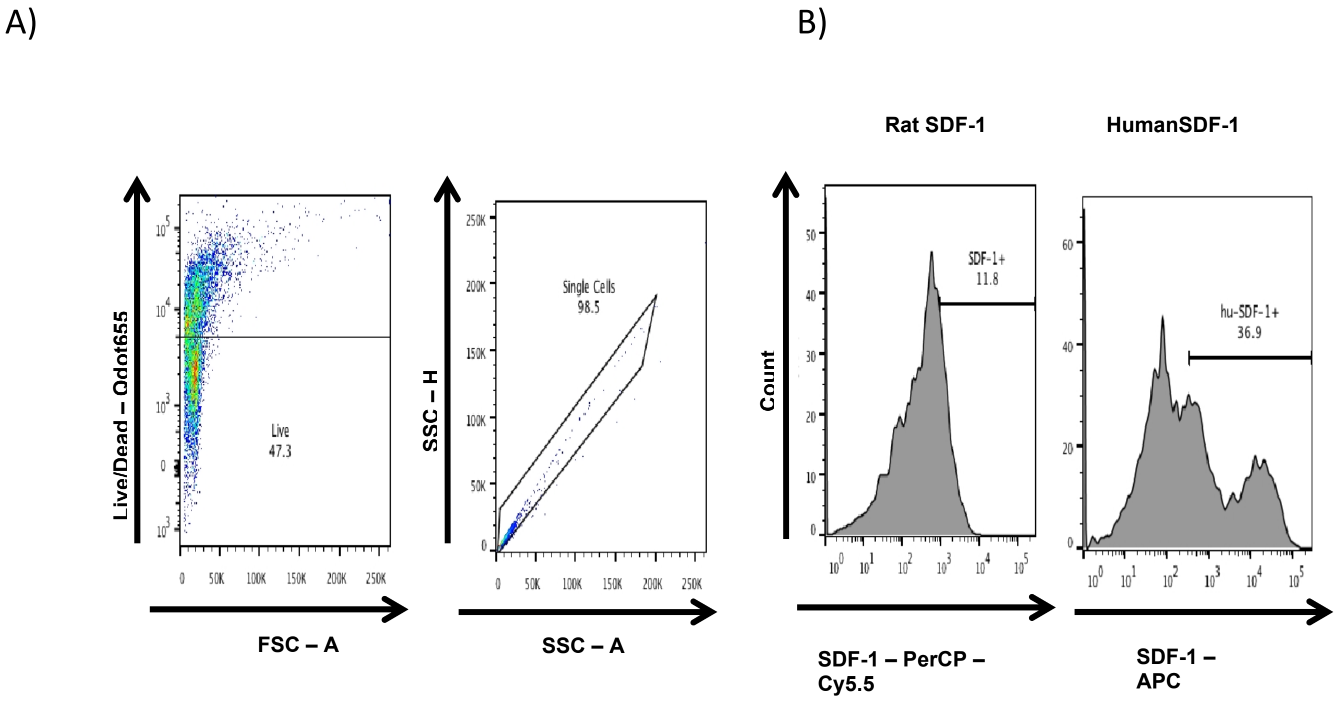

C) Gating strategy for myofibroblasts and collagen-1a α-SMA cells (Invivo experiments)

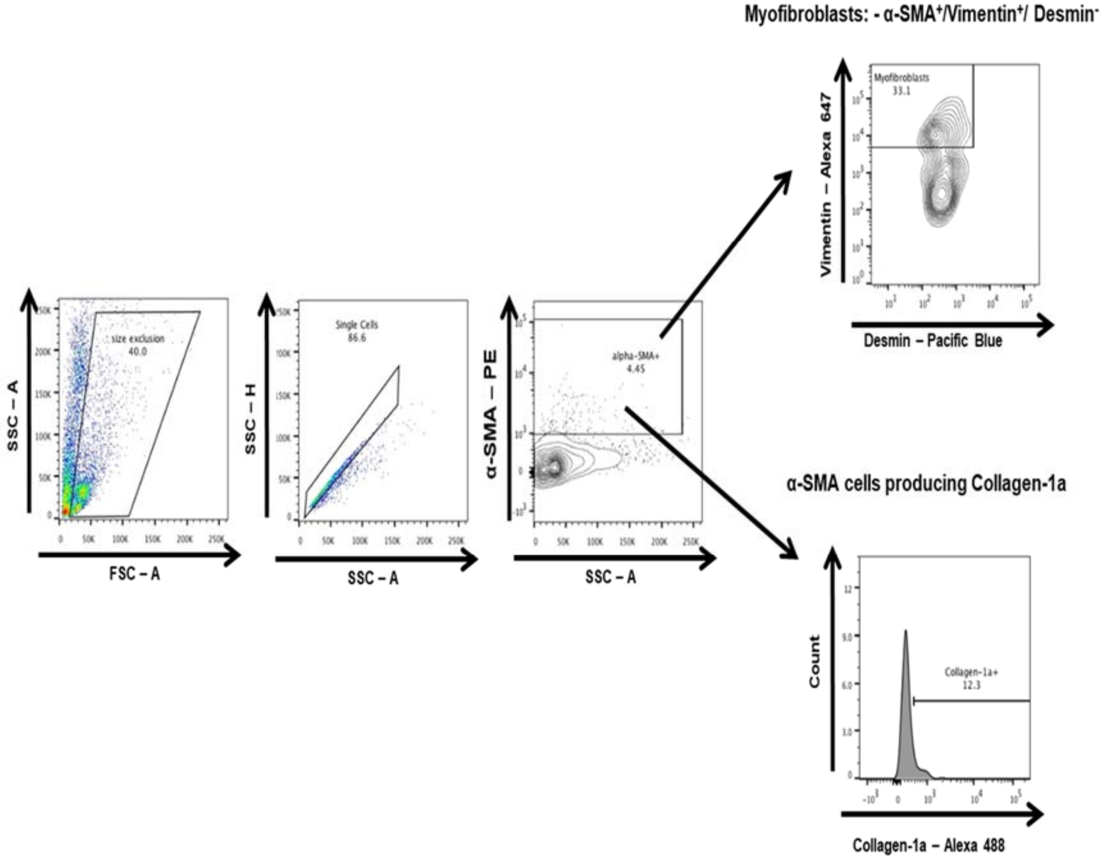

D) Gating strategy for apoptotic myofibroblasts (Invivo experiments)

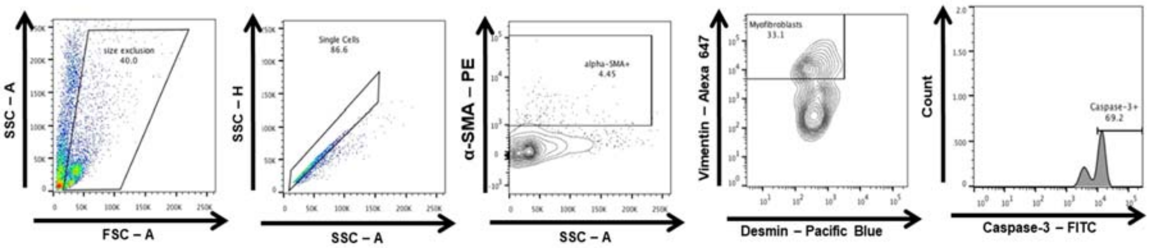

E) Gating strategy for epithelial cells and proliferating epithelial cells

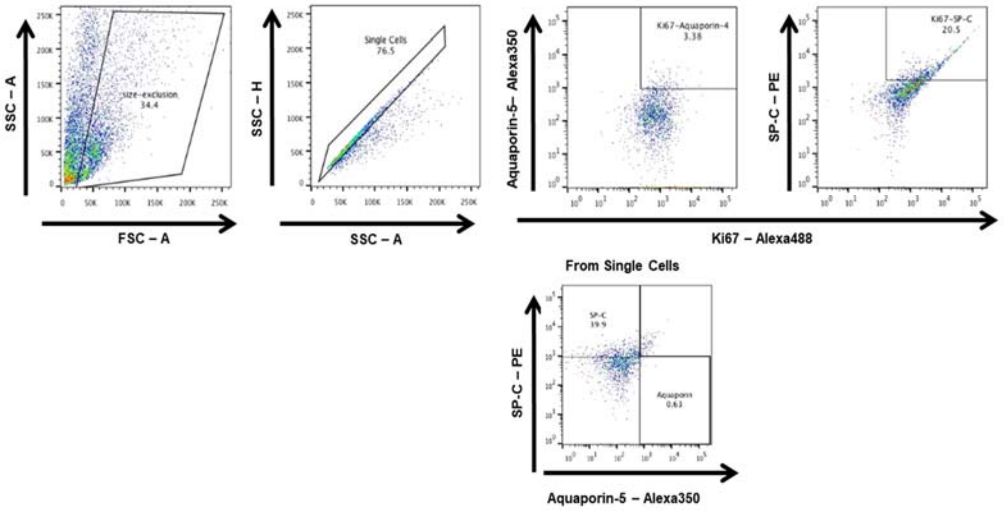

F) Gating strategy for IPF fibroblasts expressing both α-SMA and Caspase-3 (Invitro experiments)

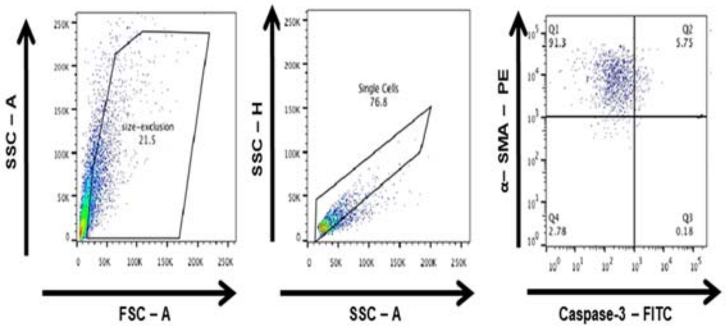

G) Gating strategy for cell specific h-SDF-1 secretion

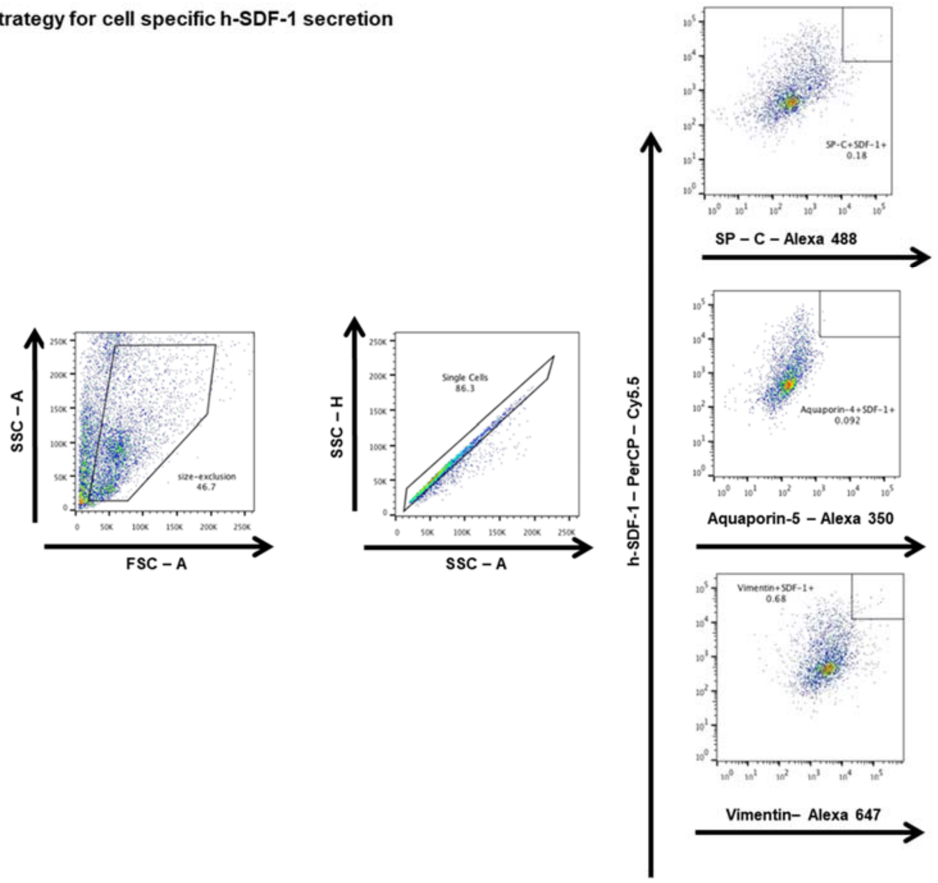

Figure S2. Flow cytometry gating strategies. Representative gating strategies for all cell types and measured end-points are shown. Lists of the antibodies that were used and technical specifications are shown on Table S1. More specifically: (A) Gating strategy for rat and human SDF-1 secreting cells. (B) Gating strategy for MSCs and CXCR4/SDF-1 expression. (C) Gating strategy for myofibroblasts and collagen-1a α-SMA cells. (D) Gating strategy for apoptotic myofibroblasts. (E) Gating strategy for epithelial cells and proliferating epithelial cells. (F) Gating strategy for IPF fibroblasts expressing both α-SMA and Caspase-3. (G) Gating strategy for cell specific hSDF-1 secretion. For all figures except (F), cells obtained from rat lung homogenates were used. For (F) cells from IPF patients were used. For each sample, at least 10,000 live, single events were recorded at an LSR-II BD FACS instrument.

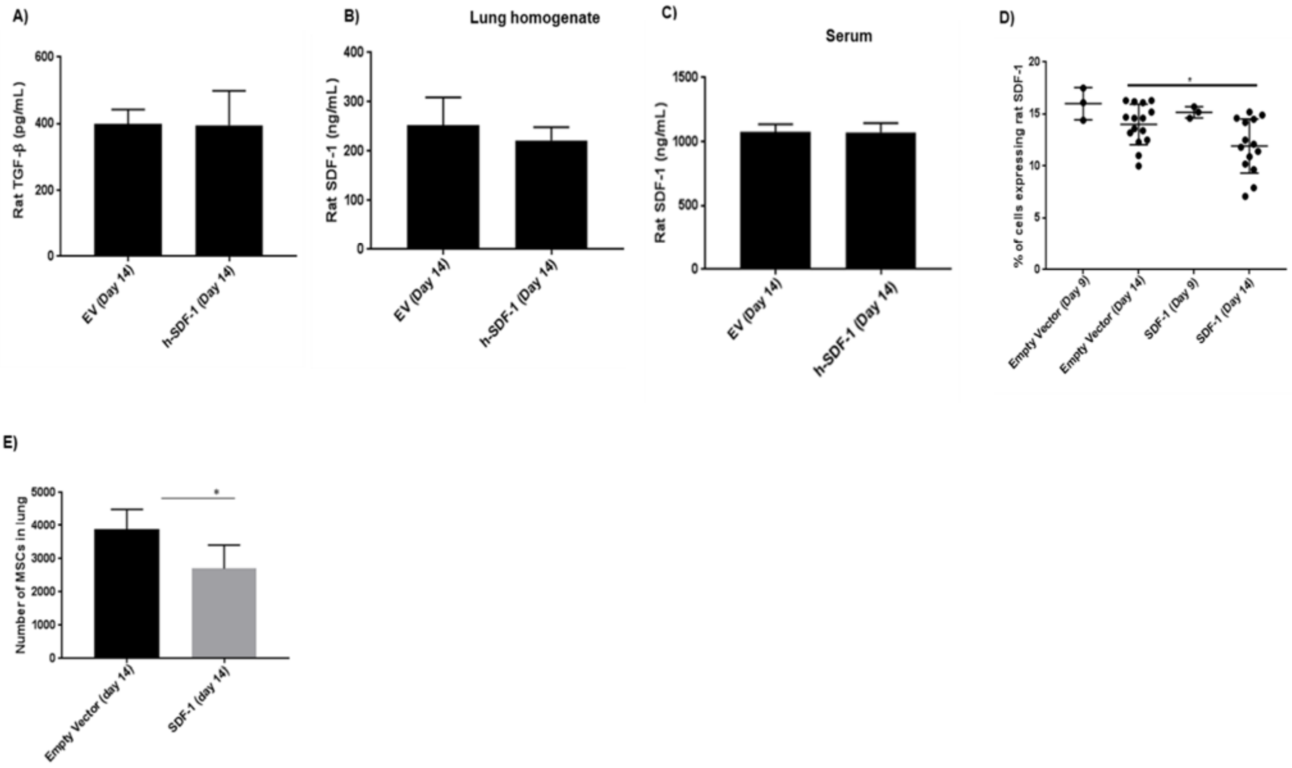

Figure S3. Rat TGF-β and rat SDF-1 measurements *in vivo*. (A) Rat TGF-β levels. (B) Rat SDF-1 levels in lung homogenates and (C) Serum. ELISA measurements were used for quantification of rat TGF-β and rat SDF-1 (n=15). No significant differences are observed. Rat SDF-1 expression (D) Percentage of cells expressing rat SDF-1 seven days after gene transfer. (E) Number of MSCs in the lung 7 days after SDF-1 gene transfer were not increased compared to the control group. * $p < 0.05$.

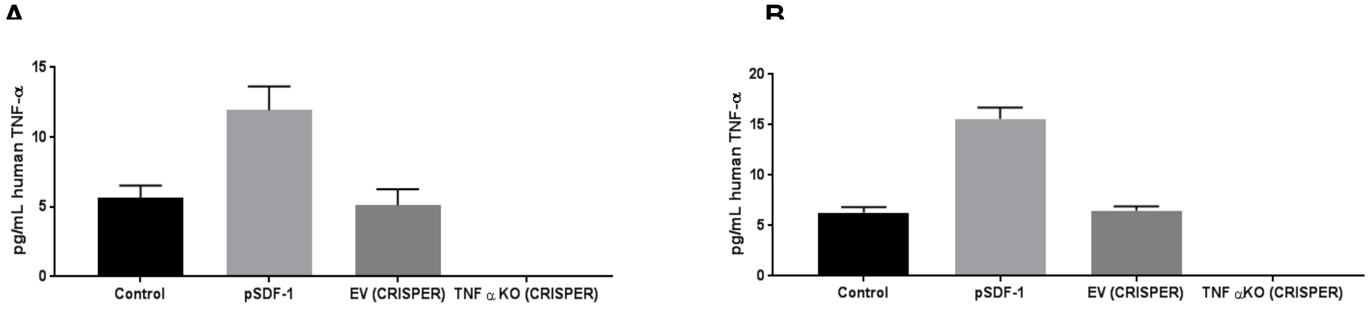

Figure S4. pSDF-1 transfection of IPF fibroblasts after TNF-α CRISPER/Cas9 KO transfection did not induce production and release of TNF-α as measured in cell culture supernatant (A) and in cell extract (B) by ELISA.

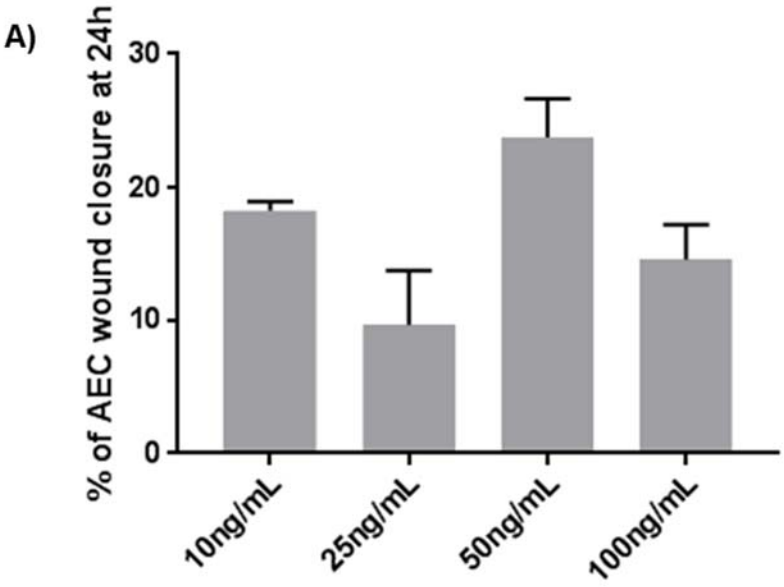

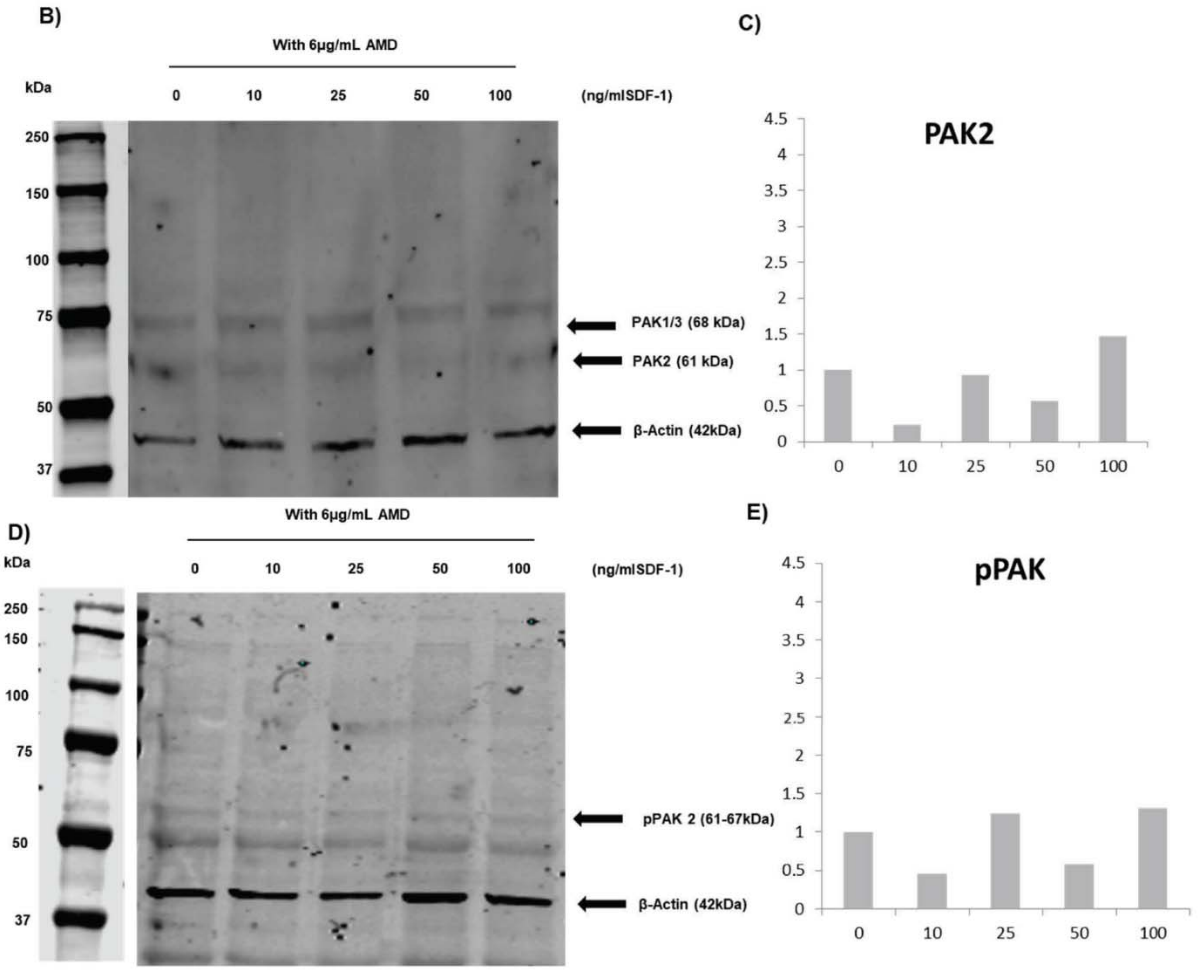

Figure S5. Inhibitory effect of AMD to SDF-1 in regards to wound healing capacity and PAK expression. For the wound healing and Western blot experiments, A549 cell cultures were treated for 20min with 6μg/mL of AMD, a CXCR4 receptor antagonist. The h-SDF-1 concentrations are shown on X-axis. Cells were initially treated with AMD and then with h-SDF-1. (A) AMD inhibits the effect of SDF-1 on epithelial cell migration. Wound Healing assay for A549 cells (n=3, Error bars: Mean±SEM). Regarding PAK expression, Western blot was performed. (B) PAK1/2/3 expression. (C) Quantification of PAK1/3 signal. (D) pPAK expression. (E) Quantification of PAK2.

Supplementary data

More specifically, a panel was used for myofibroblasts and collagen secreting cells with or without the addition of Caspase-3. Another one for cell specific (*i.e.* macrophages, Type-I, Type-II alveolar epithelial cells and myofibroblasts), while human and rat SDF-1 expression were also examined. Cells that were α-SMA$^+$/Vimentin$^+$/Desmin$^-$ were considered myofibroblasts. Cells that were α-SMA$^+$/Collagen-1a$^+$ were considered as collagen secreting α-SMA cells. Macrophages were characterized by lymphocyte gating and CD68 expression, while Type-I and Type-II alveolar epithelial cells were determined according to Aquaporin-4 and SP-C expression respectively. Finally, another antibody panel was developed to measure epithelial cell proliferation (*i.e.* SP-C, Aquaporin and Ki67) *in vivo*. Regarding *in vitro* experiments, IPF fibroblasts were analyzed for α-SMA expression and Caspase-3. For another panel, cells were stained for epithelial cell markers (*i.e.* Aquaporin-4 and SP-C) and Ki67 in order to measure epithelial cell proliferation. A vitality dye was also utilized. The entire surface and intracellular staining procedures were done in ice and the manufacturers' recommendations were followed. Antibody characteristics are shown on Table S1.

Table S1. Flow cytometry and Western blot antibodies. The list includes all antibodies that used for the flow cytometry and Western blot experiments with all technical specifications.

**Flow Cytometry**	**Clone**	**Fluorochrome**	**Isotype**	**Dilution**	**Company**	**Cat. No.**
Lineage	OX-82	Purified	IgG1	1 to 200	My Bio Source	MBS520383
CD45	OX-1	APC-Cy7	IgG1 kappa	1 to 200	Biolegend	202216
CD29	HTK888	purified	IgG	1 to 200	Biolegend	102202
CD44	OX-50	Alexa488	IgG1	1 to 200	BIO-RAD	MCA643F
CD90	OX-7	PE-Cy7	Thy-1	1 to 200	Biolegend	202518
CD105	MEM-226	PE	IgG2a	1 to 200	Acris	SM3078R
SDF-1 (hu)	79018	Purified	IgG1	1 to 50	R&D Systems	MAB350-SP
Goat anti Rabbit[1]	polyclonal	Pacific Blue	-	1 to 100	Thermo Fischer Scientific	P-10994
Anti-mouse IgG1[2]	RMG1-1	APC-Cy7	IgG	1 to 200	Biolegend	406619
Anti-mouse IgG[3]	Poly4053	PerCP-Cy5.5	IgG	1 to 200	Biolegend	405314
Live/Dead	-	Qdot655	-	-	Thermo Fischer Scientific	L10120
SDF-1 (Rat)	79018	APC	IgG1	1 to 200	NOVUS	IC350A
SP-C	polyclonal	PE	IgG	1 to 100	Bioss	bs-10067R-PE
Aquaporin-4	polyclonal	Alexa 350	IgG	1 to 50	Bioss	bs-0634R-A350
Ki67	-	purified	-	-	abcam	ab15580
Goat anti Rabbit[4]	polyclonal	Alexa 488	IgG	1 to 2000	abcam	ab150077
Caspase-3	-	FITC	-	-	BD	550480
CD68	FA-11	PE	IgG2a	1 to 100	Biolegend	137013
SP-C	polyclonal	Alexa 488	IgG	1 to 100	Bioss	bs-10067R-A488
Aquaporin-4	polyclonal	Alexa 350	IgG	1 to 50	Bioss	bs-0634R-A350
Vimentin	EPR3776	Alexa 647	IgG	1 to 5000	abcam	ab194719
Anti-mouse IgG[5]	Poly4053	PerCP-Cy5.5	IgG	1 to 200	Biolegend	405314
**Western Blot**						
PAK1/2/3	polyclonal	-	-	1 to 1000	Cell Signaling Technology	2604
Phospho-PAK1/PAK2	polyclonal	-	-	1 to 1000	Cell Signaling Technology	2605
B-Actin	Mouse monoclonal	-	IgG2b	1 to 1000	LI-QOR	926-42212
Mol. Weight marker	-	-	-	-	LI-QOR	928-40000
Goat anti Rabbit IgG[6]	polyclonal	IRDye-680RD	IgG	1 to 10000	LI-QOR	926-68074
Goat anti Mouse IgG[7]	polyclonal	IRDye-800RD	IgG	1 to 5000	LI-QOR	926-32211

[1]: Secondary antibody to CD29

[2]: Secondary antibody to CD45

[3]: Secondary antibody to human SDF-1

[4]: Secondary antibody to Ki67

[5]: Secondary antibody to human SDF-1

[6]: Secondary antibody to β-actin

[7]: Secondary antibody to PAKs
